# Leveraging the electronic health records to mitigate the effects of a nation-wide shortage of blood culture bottles

**DOI:** 10.1093/jamiaopen/ooag003

**Published:** 2026-01-27

**Authors:** Dean Scott Miner, Gregory Knapp, Kristen Lambert, Kenneth Brabble, Christopher R Dennis, John Markantonis, Jacob Pierce, John Hanna, Richard J Medford

**Affiliations:** IS Services, East Carolina University Health, Greenville, NC 27834, United States; Department of Pediatrics, ECU Brody School of Medicine, Greenville, NC 27834, United States; IS Services, East Carolina University Health, Greenville, NC 27834, United States; Department of Family Medicine, ECU Brody School of Medicine, Greenville, NC 27834, United States; IS Services, East Carolina University Health, Greenville, NC 27834, United States; IS Services, East Carolina University Health, Greenville, NC 27834, United States; IS Services, East Carolina University Health, Greenville, NC 27834, United States; Department of Pathology, ECU Brody School of Medicine, Greenville, NC 27834, United States; IS Services, East Carolina University Health, Greenville, NC 27834, United States; Department of Internal Medicine, ECU Brody School of Medicine, Greenville, NC 27834, United States; IS Services, East Carolina University Health, Greenville, NC 27834, United States; Department of Internal Medicine, ECU Brody School of Medicine, Greenville, NC 27834, United States; IS Services, East Carolina University Health, Greenville, NC 27834, United States; Department of Internal Medicine, ECU Brody School of Medicine, Greenville, NC 27834, United States

**Keywords:** shortage, alternatives, clinical decision support, EHR optimization, data analytics, blood culture, supply chain, resource optimization

## Abstract

**Objective:**

To evaluate how electronic health records (EHR) optimization and data analytics supported a large rural health system action plan to mitigate the effects of a nation-wide blood culture bottle shortage.

**Materials and Methods:**

Following the announcement of a nationwide blood culture bottle shortage on July 10, 2024, we implemented EHR order modification, alternative alerts (LMAs) for clinical decision support (CDS), and developed a data analytics dashboard to track daily orders and inventory. We analyzed changes in daily blood culture specimen utilization before and after the EHR interventions using run charts. We assessed blood culture positivity and contamination rates, and provider interactions with LMAs as process measures.

**Results:**

The EHR-based interventions led to a sustained 81% reduction in daily blood culture utilization during the shortage. Blood culture contamination rates remained consistent at 5.1% pre- and post-interventions, and positivity rates were stable (13.5% pre vs 12.8% post). Analysis of LMAs showed that 16% of blood culture orders were canceled after the alert, with only 3% reordered within one hour. The utilization and inventory monitoring reports became top 10% most-used within the health system, supporting operational decisions.

**Discussion:**

Combining EHR optimization, CDS via LMAs, and data analytics effectively mitigated a critical resource shortage, demonstrated by a sustained 81% reduction in blood culture bottle utilization without compromising patient care. EHR order modifications contributed to the initial reduction; LMAs sustained the decrease and were well-received compared to traditional alerts. Rapid development of data analytics reports supported data-driven operational decisions.

## Introduction

The optimization of Electronic Health Records (EHRs) has become critical for managing healthcare resources, especially during times of supply shortage. Through the use of Clinical Decision Support (CDS) tools, EHRs can enhance clinical workflows by reducing unnecessary tests, improving resource allocation, and maintaining patient safety. In July 2024, ECU Health, a large academic rural health care system based in eastern North Carolina, leveraged the EHR to adapt to an emerging nationwide shortage of blood culture bottles, a critical diagnostic resource used for identifying bloodstream infections. This shortage necessitated immediate action to ensure continued patient care while conserving limited supplies.

EHR based Clinical Decision Support (CDS) tools have proven effective in optimizing diagnostic test orders in various healthcare settings.^1–4^ For example, studies have shown that CDS interventions can reduce unnecessary imaging^4^ and laboratory tests,^1^^,^^2^ improving adherence to clinical guidelines^5^^,^^6^ while minimizing resource overuse.^6^^,^^7^ Specifically, in the context of diagnostic stewardship, CDS tools have demonstrated success in reducing test overutilization without compromising diagnostic accuracy.^1^^,^^7–10^

In emergency settings, CDS that provide real-time prompts at the point of care have been shown to influence clinician behavior and improve adherence to evidence-based practices.^11–13^ EHR optimization coupled with CDS have not only improved diagnostic stewardship but also yielded economic benefits. Several studies have reported cost savings from reduced laboratory test orders, such as unnecessary lab testing or transfusion practices, which highlight the potential financial impact of CDS interventions.^2^^,^^7^^,^^14–16^

Reacting to a recent nationwide supply shortage for blood cultures, many institutions implemented diagnostic stewardship CDS to urgently mitigate the blood culture supply issues by restricting the ordering of blood cultures.^17–19^ Furthermore, we leveraged analytics, in combination with CDS, to provide valuable insights that allow our organization to proactively manage limited supplies without compromising patient safety.

This case study aims to examine how the combination of EHR optimization, deployment of CDS tools, and analytics supported a large rural health system’s response to a nation-wide blood culture shortage. By assessing the effects of these interventions on blood culture utilization, this paper provides a framework for other healthcare organizations to leverage the EHR and analytics to manage similar resource shortages effectively, ensuring both operational efficiency and patient safety.

## Materials and methods

ECU Health is a large rural health system, which includes 9 rural hospitals, serving 1.4 million patients across 29 counties. To mitigate the effects of a nation-wide blood culture shortage, we focused on EHR optimization, CDS tools, and data analytics solutions. We report tracked blood culture utilization over a duration of 8 months: comparing four months prior to and four months after the blood culture shortage was announced on July 10, 2024.

**Figure 1.**
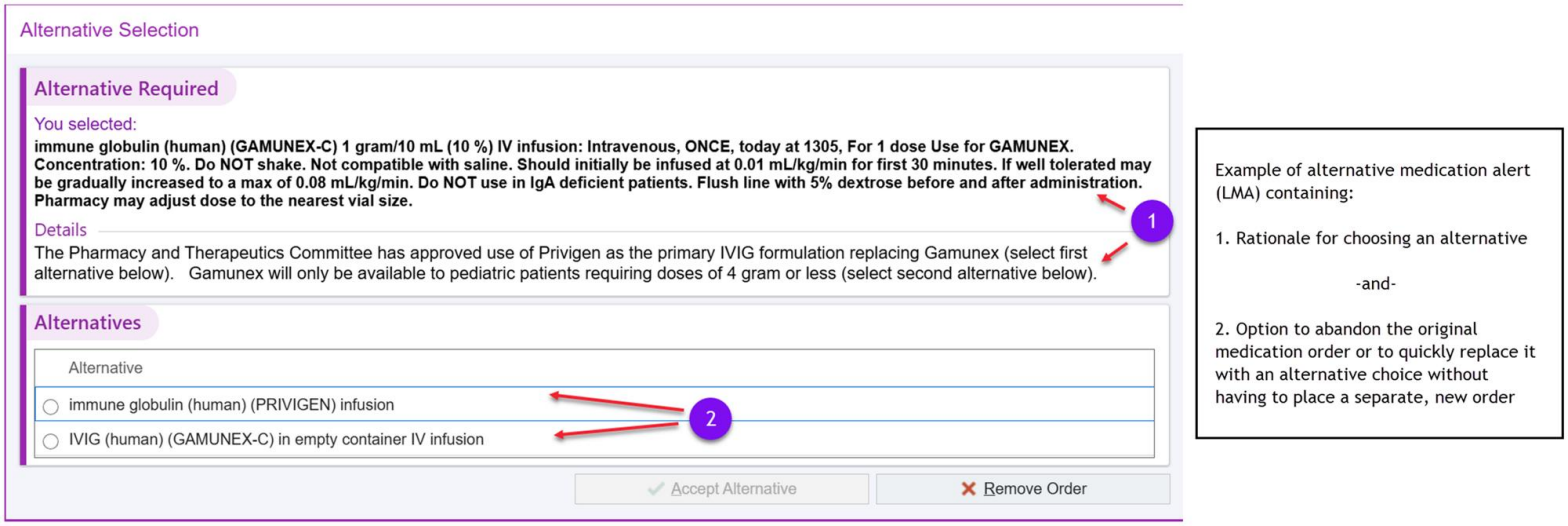
Example of alternative medication alert. © 2026 Epic Systems Corporation.

**Figure 2.**
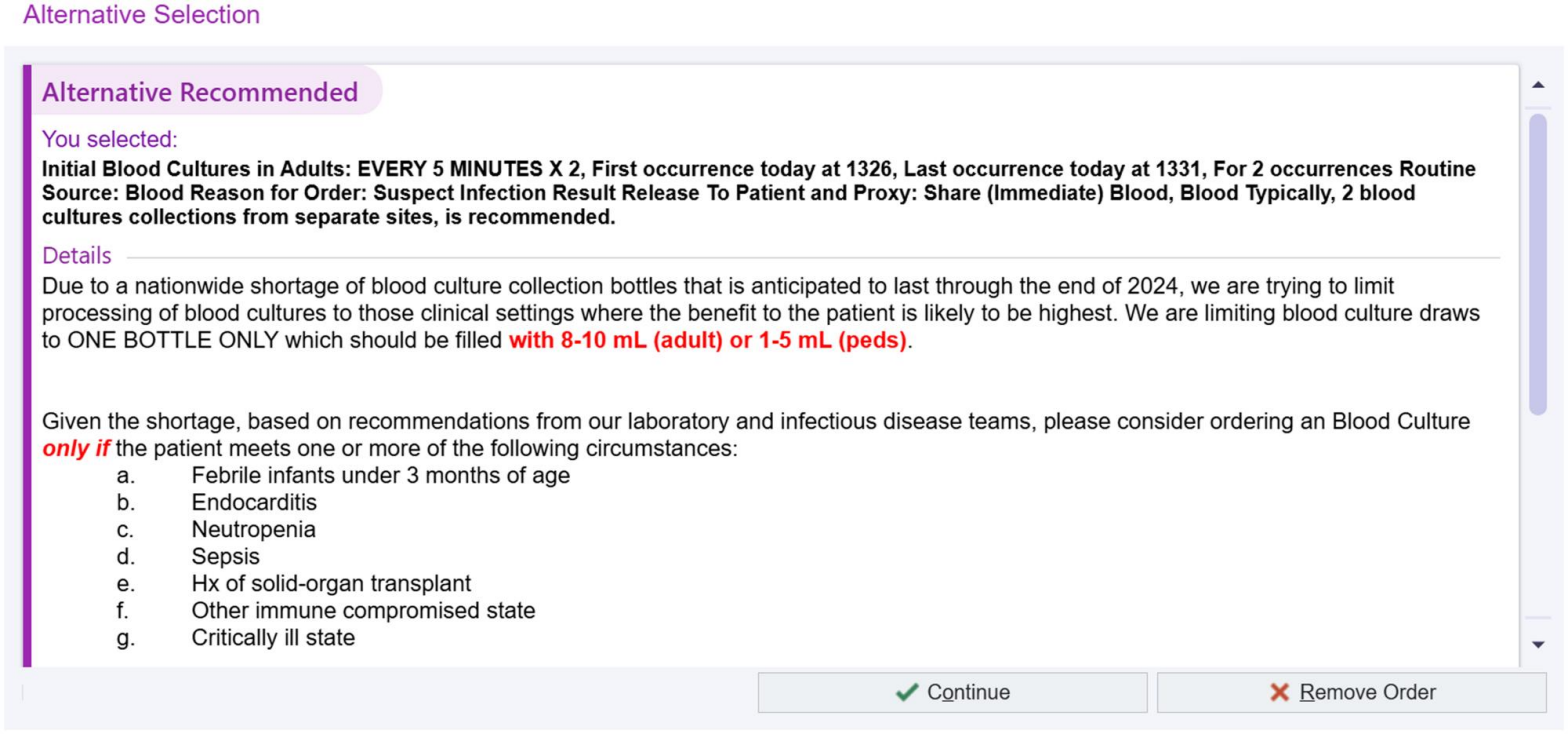
Repurposed alternative alert for non-medication (blood culture) order. © 2026 Epic Systems Corporation.

Two primary EHR interventions were implemented to address the blood culture shortage across the ECU health system’s hospital network focusing on the health system utilization and ordering patterns in the face of the shortage:

1. EHR Order Modification (July 18, 2024): The default order for blood cultures was modified from two sets (four bottles total: two aerobic and two anaerobic) to a single aerobic bottle. This change was made to immediately reduce the number of blood culture bottles used per order. The change in the blood culture order resulted in a printout of a single label designed to be affixed to a single blood culture bottle after collection by the nurse and also resulted in placement of a restriction on the end-user’s ability to place another blood culture order within 48 hours following the original order placement.
2. Clinical Decision Support Tool—deployment of alternative alerts (LMA) (July 29, 2024): LMA is a programming option to offer in-line CDS to clinicians who have placed an order for a specific medication or procedure. LMAs are frequently used when the health system wishes to re-direct the ordering clinician to consider an alternative therapy or choice, because of new evidence-based guidelines, patient safety concerns, or transient medication shortages. LMAs are inserted into the ordering workflow when the patient meets a criterion or a clinically relevant rule, prompting the clinician to select an alternative option or to proceed with the original order as depicted in Figure 1. LMA programming architecture permits the delivery of a window of explanatory text, with optional links to other reference material, followed by an offer to continue with an alternative order in a single click or to continue with the original order if appropriate. We leveraged the LMA option for CDS in an innovative way, for a non-medication-based order to provide in-line guidance to clinicians when they have decided to place an order for a blood culture (Figure 2). We also implemented LMAs as additions to existing blood culture orders for aerobic, anerobic, fungal and non-blood (tissue) culture orders in the system. We decided to deploy the LMA on both traditional blood culture orders (aerobic, anerobic and fungal) in addition to non-blood (tissue) culture orders understanding end-user behavior which historically has led some clinicians to select the incorrect order. For our blood culture LMAs, the “alternative” to the original order placement was to cancel the original order, while for the non-blood culture LMA, the “alternative” presented to the clinician was to select an actual blood culture order in lieu of the incorrect non-blood culture order, or to cancel the request for a culture altogether if not clinically appropriate (Figure 2).

The primary value of the LMA was to deliver clinical guidance reminding clinicians about medically appropriate indications for drawing a blood culture along with a concrete reminder that if the decision is made to proceed, the clinician should draw an appropriate volume of blood and place it in a single bottle to conserve the limited resource. The deployed series of LMAs were designed to encourage clinicians to limit unnecessary tests while allowing the flexibility to proceed with the order if clinically justified. Combined with a primary restructure of the actual blood culture orderables to limit label printing for one bottle, these interventions were part of an operational action plan aimed at balancing clinical needs with resource conservation during the shortage.

Additionally, we developed a Microsoft Fabric-based analytics dashboard to monitor the blood culture utilization and the system’s inventory. The dashboard provided daily updates on the number of blood culture bottles ordered, specimens collected, and the remaining inventory of aerobic, anaerobic, and pediatric blood culture bottles. These metrics were closely tracked by health system operational and clinical leadership teams to ensure that inventory levels remained manageable during the shortage and to support data-driven decision making.

The primary outcome measure we report is the change in daily blood culture orders and specimen utilization before and after the EHR interventions. This is visualized using a run chart, capturing key intervention dates, and the resulting trends in blood culture bottle use. Furthermore, we captured the following process measures to reflect the indirect effects of these interventions: blood culture positivity rates, blood culture contamination rates (to evaluate whether reducing the number of collected bottles from four to one increases contamination or inaccuracies in blood culture results), and provider interactions with the LMAs including order cancelation rate and blood culture reorders within one hour post cancelation.

## Results

**Figure 3.**
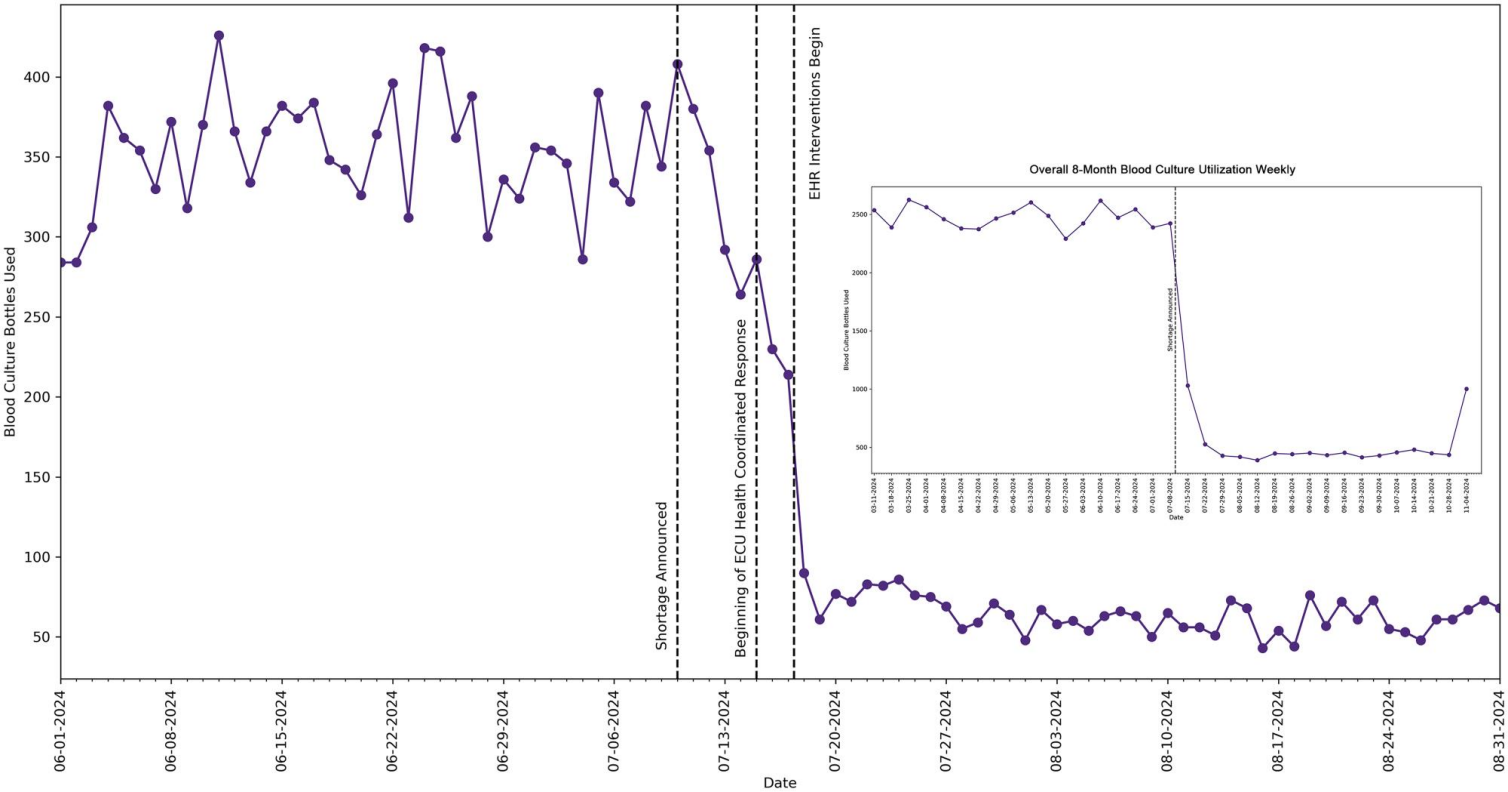
Blood culture bottle utilization pre and post intervention.

During the reported period, a significant reduction in blood culture bottle utilization was observed following the EHR-based interventions. Figure 3 shows the daily blood culture bottle usage over a run chart, highlighting key intervention points and their effects on usage trends.

Before the announcement of the blood culture shortage, daily usage fluctuated between 300 to 400 bottles per day. Between July 15, 2024, and July 17, 2024, health system leaders broadcast messages to clinicians across the system, which led to a reduction in average daily blood culture bottle utilization to around 250 bottles per day. With the implementation of the first EHR change which limited and redirected almost all blood culture orders to collections in a single aerobic bottle on July 18, 2024, our system saw a significant reduction in daily blood culture bottle utilization to around 80 bottles/day on average (an approximate 73%-80% reduction overall). After the subsequent introduction of the LMA clinical decision support tool on July 29, 2024, a further decline in utilization was noted to a range of 50-75 bottles/day on average (an approximate 80%-83% reduction overall), with the daily consumption rate remaining in this range for the remainder of the observation period till early November 2024 when the health system allowed a printout of 2 labels (one for aerobic and one for anaerobic bottle) from each order in response to the inventory improvement trends.

Blood culture contamination rate in the pre-shortage 4-month period averaged at 5.1% (537/10 486 sets). In the 4-month period post shortage, the blood culture contamination rate continued to average at 5.1% (224/4398 sets), implying that the reduction from four bottles to only one bottle did not result in an increase in the risk for contamination. Blood culture positivity rate was 13.5% in the four months pre-shortage, and 12.8% in the four months post-shortage.

**Figure 4.**
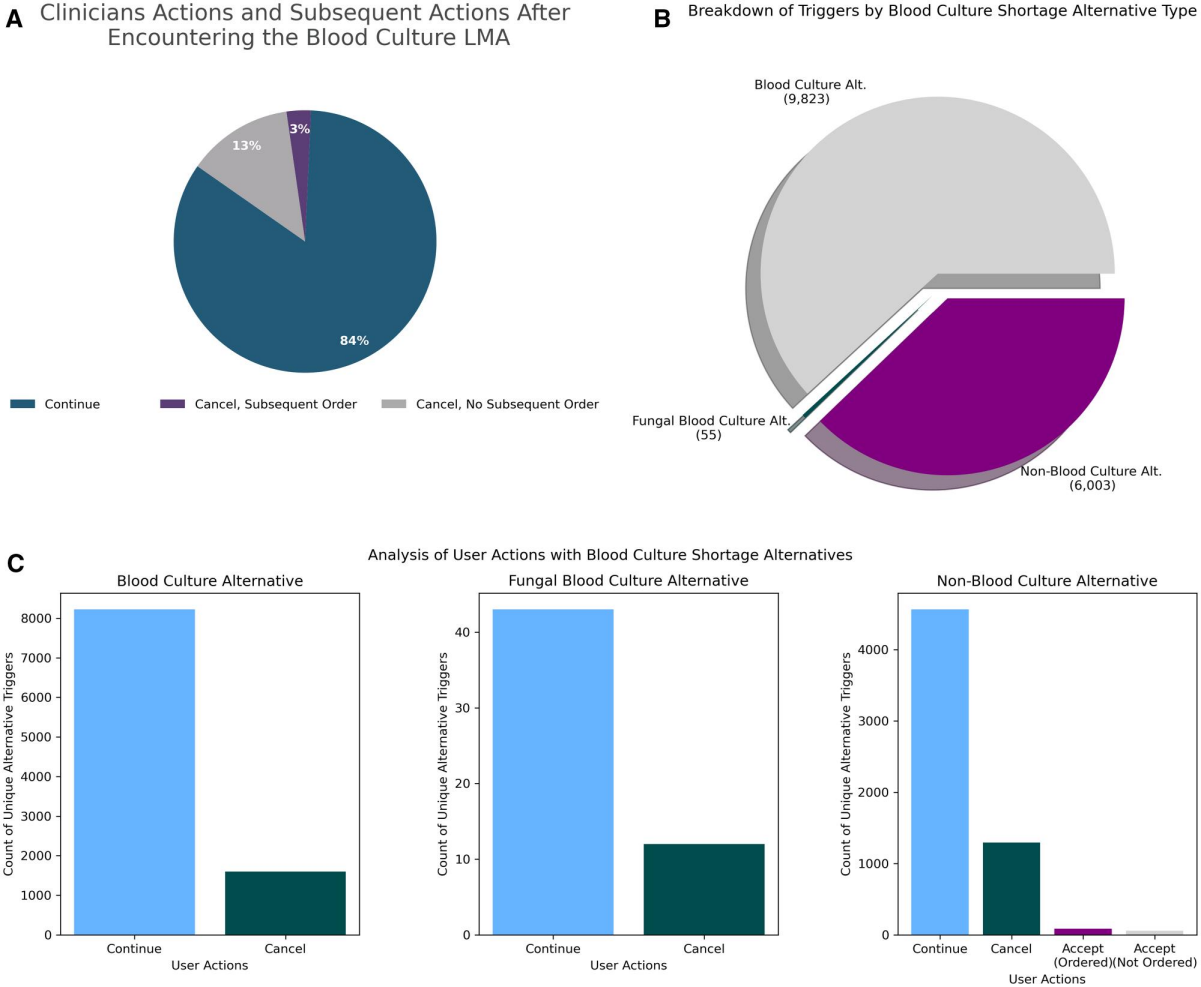
Clinician actions after exposure to Blood Culture LMA.

For the observed duration from the point of the implementation of our first EHR change on 07-18-2024 until the end of the reported period, we observed the initial placement of a combined total of 15 881 orders for either aerobic blood cultures, fungal blood cultures, or non-blood cultures. More specifically, clinicians at ECU Health attempted to place 9823 aerobic blood culture orders, 6003 non-blood culture orders, and 55 fungal blood culture orders (Figure 4). As previously described, the blood culture and the blood culture for fungus orders with their associated LMAs only offered clinicians the option to either continue with the original order or to cancel it. After being presented with these options in the LMAs displayed in the standard aerobic blood culture order, clinicians opted to continue with the original order 8223 times while canceling it 1600 times for an overall blood culture order preservation rate of 84% and cancelation rate of 16%. For fungal blood cultures, clinicians continued with the original order 43 times while canceling it 12 times, suggesting the LMA was associated with an overall blood culture order preservation rate of 78% and cancelation rate of 22%. By contrast, the LMAs built into the non-blood culture order did provide additional choices. Therefore, clinicians were able to either continue with the original order, cancel the order, or accept one of the choices to order an actual aerobic blood culture or a fungal blood culture. Given the construct of the LMA, our data collection allowed us to discriminate between clinicians who chose one of three options:

1. Those who proceeded with the original non-blood culture order = 4563 of 6003 orders (Suggesting correct order placement rate of 76%)
2. Those who selected an alternative order and then clicked “accept” = 88 of 6003 orders (Suggesting correct order re-direct rate for LMA of 1%)
3. Those who did not select an alternative order and then clicked “accept” = 58 of 6003 orders (Suggesting incorrect order/abandonment rate for LMA of 1%)
4. Those who completely canceled the non-blood culture order = 1294 of 6003 orders (Suggesting that LMA stop non-blood culture order rate of 22%)

Clearly, clinical decision making is often a dynamic process, so while a clinician may encounter one of the alternatives at a single point in time and make a choice, they may change their mind and encounter the same alternative additional times before ultimately signing an order. Understanding this possibility, we analyzed the alternative data looking for scenarios that implied the clinician accepted the guidance of the alternative but ultimately reversed course upon subsequent encounters with the CDS (Figure 4). For the blood culture alternative, of the 1600 times the clinician chose to cancel the order, a clinician subsequently chose to continue with the blood culture order within one hour in 3% of the cases. For the blood culture for fungus alternative, there were no times where the clinician subsequently chose to continue with the order out of the 12 times it was originally canceled. For the non-blood culture alternative, out of the 1440 times the clinician either accepted an alternate choice or canceled the order, 491 subsequent triggers resulted in a continuation of the original order and 16 subsequent triggers resulted in the outcome of accepting and ordering one of the alternate orders within one hour post the first LMA.

**Figure 5.**
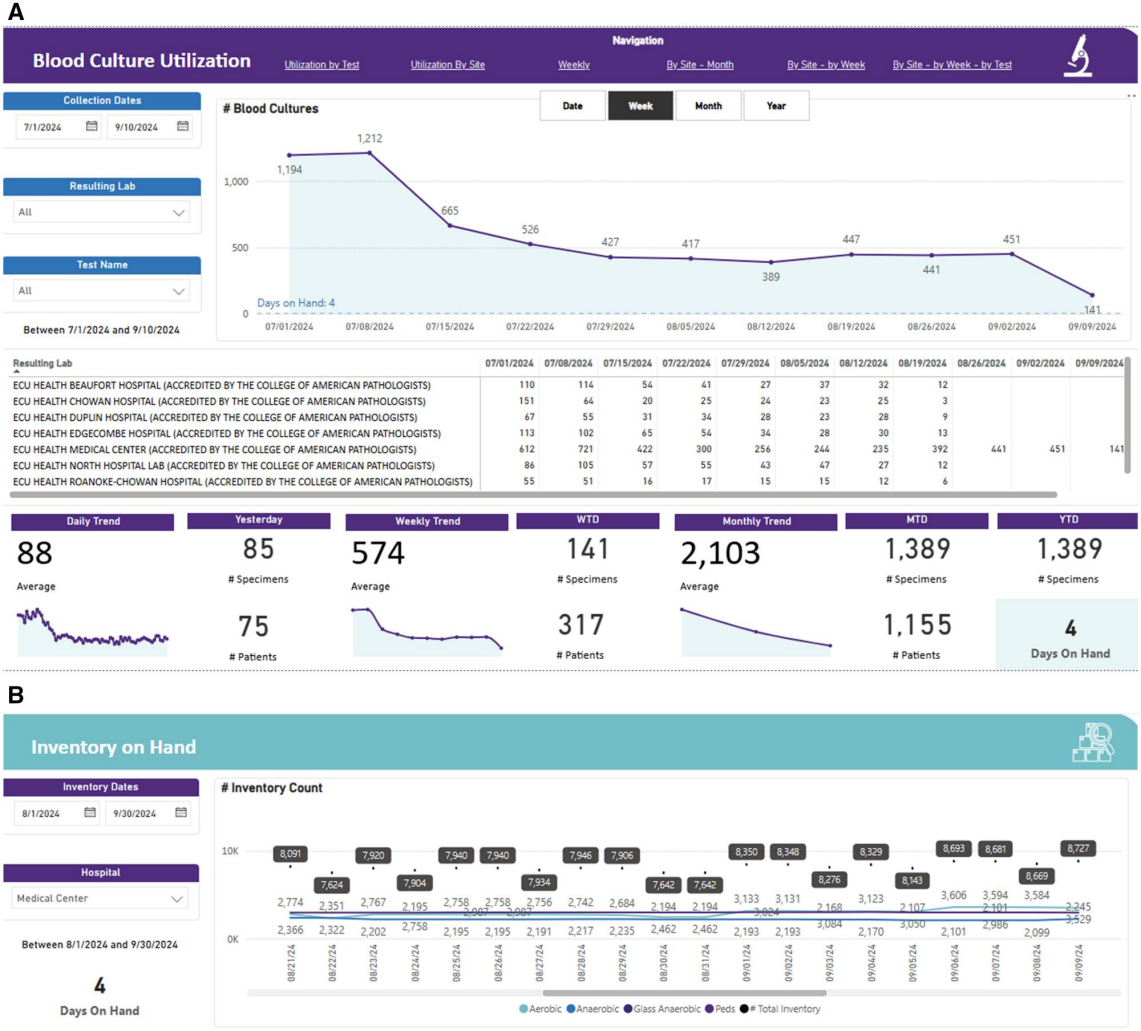
ECU Health Data Analytics Dashboards for A - Blood Culture Utilization Rates and B- Blood Culture Bottle inventory on hand.

The data analytics dashboard (Figure 5), powered by Microsoft Fabric’s end to end analytics capabilities, allowed continuous daily monitoring of blood culture bottle use and inventory levels. This tool enabled the health system to adjust its operational response and prevent complete consumption of our blood culture bottle supply during the shortage. One of the dashboard’s components was an inventory tracker that effectively monitored ongoing aerobic, anaerobic, and pediatric blood culture bottle inventory. No critical shortages were reported during the defined period, and stock levels were kept within acceptable limits. The blood culture shortage report was ranked in the top 10% of reports used among all ECU Health reports in Power BI and supported data-driven operational decision making through the shortage period.

## Discussion

Our reported observations demonstrated that a combination of EHR optimization, CDS, and data analytics to empower decision making can significantly mitigate the effects of a critical resource shortage, such as the nation-wide blood culture shortage faced by our health system. The implementation of targeted interventions, including the modification of blood culture order defaults and repurposing LMAs for non-medication clinical decision support purposes, resulted in a sustained 81% reduction in daily blood culture bottle utilization. While many clinical decision support interventions require end-users to navigate to other areas of the chart to obtain the information necessary to address the alert, the LMAs that were deployed here were constructed in a way that allowed clinicians to understand why they were being interrupted and take immediate action to minimize disruptions to the workflow. The LMA CDS intervention seemed to be well received by clinicians based on subjective feedback from end-users. Clinicians also seemed to understand that the CDS was necessary to conserve a limited but critical resource, likely limiting complaints related to alert fatigue. In addition, these changes allowed the health system to maintain clinical operations with minimal disruption to patient care.

The immediate effects of the interventions were evident within days of their implementation. The change in the default order (from two sets of blood cultures to a single aerobic bottle) led to a rapid and substantial decline in utilization, which was further reinforced by the clinical decision support tool in the form of an LMA. This demonstrates the power of EHR-driven interventions in influencing clinician behavior, particularly in situations where resource conservation is critical. Furthermore, the sustained reduction in utilization highlights the potential for these tools to have lasting effects beyond the initial crisis.

Although there are limited published studies^17^^,^^18^ addressing the specific use of EHR optimizations to manage blood culture shortages, the results are consistent with broader literature on the role of CDS in promoting evidence-based practice and reducing unnecessary testing. This end use case of alternative alerts represents a practical and efficient solution for health systems facing unexpected shortages or operational challenges, if those systems have the appropriate expertise and adequate resources to do the required EHR build. In our instance, we chose an LMA over a more interruptive traditional Our Practice Advisories (OPA) because it allowed clinicians to proceed with their usual ordering practices (no need for re-education) while presenting them with in-line education and in-line alternative order options when available during the ordering process, increasing the probability of achieving the desired alteration in clinician behavior.

The use of alternatives helped to sustain our blood culture bottle conservation strategy in concert with other EHR changes and strong organizational buy-in. Subjectively, clinicians have responded favorably when presented with LMAs during the ordering process and we have since revised some of the verbiage in the LMAs since the wording may have overly deterred some clinicians from ordering blood cultures when they may have been appropriate based on clinical circumstances. Overall, the data seems to suggest that our blood culture order LMA “successfully” prompted 16% of the originally placed orders to be canceled, presumably because the LMA guidance helped the ordering clinician to understand that they were not medically indicated based on recommendations from our antimicrobial stewardship team. Given the critical nature of the blood culture bottle shortage, this intervention was clearly an important contribution to the conservation effort. Going forward, there is an opportunity to enhance these analytics further to establish the net effect at the patient level and to expand the use of LMAs for other clinical situations where in-line delivery of CDS would be desirable to modify clinician ordering behaviors at the time of order placement.

One of the other key strengths of the coordinated response lies in the combination of daily analytics reports along with the EHR optimizations, providing a comprehensive tracking solution to both operational and clinical challenges. The use of a dashboard to track blood culture usage and inventory allowed for dynamic adjustments in resource management, ensuring that the system could adapt as the situation evolved.

While the EHR interventions were highly effective, several challenges were encountered during the optimization process: (1) There are some settings used to configure LMAs that are shared between medication and procedure order use cases. These settings limited some of the options at our disposal for developing a workflow that was intuitive to users. For example, configuring the LMA to allow for overriding the guidance, while requiring a reason for doing so, led to conflicts since some of the options included reasons such as "Refill authorization" which would not make sense in the context of the blood culture bottle shortage. Removing the option would negatively impact the scenarios where this choice is appropriate though. Therefore we opted to configure the LMAs for the blood culture bottle shortage in a way that avoided reliance upon this list of values entirely. (2) While the reduction in blood culture usage was substantial, the clinical implications of drawing only one aerobic bottle instead of the traditional four-bottle set remain unclear. Although our blood culture positivity rates and blood culture contamination rates stayed unchanged throughout, and no immediate safety concerns were reported despite reducing the number of bottles by 75%, our hypothesis was that we would be at higher risk for blood culture bottle contamination. Hence, there is clearly a need for further research to determine the long-term clinical impact of this practice, particularly with regard to diagnostic accuracy and patient outcomes.

Moving forward, several areas warrant further exploration: (1) Additional studies are needed to assess the impact of reduced blood culture use on patient outcomes, particularly in terms of diagnostic delays or missed infections. (2) The success of this intervention suggests that similar EHR optimizations could be applied to other areas of resource management, such as antibiotic stewardship or imaging orders. (3) The limitations of the current out-of-the-box LMA set-up—particularly by refining the override options—could further enhance the utility of LMA use in resource-limited settings.

## Conclusion

This study highlights the effectiveness of the combination of EHR order modification, LMA, and data analytics in managing healthcare resource shortages. By modifying blood culture electronic orders and maximally leveraging LMA for clinical decision support, our health system significantly reduced blood culture utilization and avoided critical shortages, with minimal anticipated impact on patient care as demonstrated by our sustained blood culture positivity and contamination rates pre and post intervention. Rapidly developed data analytics solutions at the time of shortage supported urgent operational data-driven decision making during an evolving critical situation.

## Data Availability

Project data available upon request.
